# The role of mobility in sexual risk behaviour and HIV acquisition among sub-Saharan African migrants residing in two European cities

**DOI:** 10.1371/journal.pone.0228584

**Published:** 2020-02-05

**Authors:** Sónia Dias, Ana Gama, Jasna Loos, Luis Roxo, Daniel Simões, Christiana Nöstlinger

**Affiliations:** 1 NOVA National School of Public Health, Public Health Research Centre & Global Health and Tropical Medicine, Universidade NOVA de Lisboa, Lisboa, Portugal; 2 Comprehensive Health Research Centre (CHRC), Lisboa, Portugal; 3 Department of Public Health, Institute of Tropical Medicine, Antwerp, Belgium; 4 Grupo de Ativistas em Tratamentos, Lisboa, Portugal; Agencia de Salut Publica de Barcelona, SPAIN

## Abstract

**Background:**

Migrants from high endemic countries accounted for 18% of newly diagnosed HIV infections in Europe in 2017. Knowledge on the link between HIV risk and post-migration travels and their impact on HIV acquisition is scarce, but critical to inform prevention. This study aims to explore risky sexual behaviour and HIV-acquisition among sub-Saharan African migrants, and to assess post-migration mobility as a determinant of sexual risk behaviour.

**Methods:**

Data from two cross-sectional bio-behavioural surveys to assess HIV-prevalence conducted in Lisbon and Antwerp were analysed to explore migration-related characteristics, travel patterns, and sexual risk taking in the host country and abroad. Bi- and multivariate associations were estimated through adjusted odds ratios and 95% confidence intervals; multivariable logistic regression determined factors associated with condomless sexual intercourse.

**Results:**

Among N = 1508 participants above 18 years (58% males), 68% travelled post-migration (49.2% reported intercourse abroad). The overall proportion of condomless sex at last sexual intercourse was high (68.1%). The odds of condomless sex in the host country was five times higher when the last sexual intercourse abroad was also condomless [OR:5.32; 95%CI:2.98–9.25]. About half of the travellers reported concurrency, i.e. a regular partner in the host country while having other sexual partners abroad. Almost three percent of the participants reported being HIV+, but 5% had a reactive HIV test-result, with similar proportions among travellers and non-travellers. Also, among the n = 75 participants with reactive HIV test-results, condomless sex occurred (n = 40) and was associated with mobility.

**Conclusions:**

Sub-Saharan African migrants are mobile and engage in sexual risk behaviours in the countries of residence and while travelling, increasing risk of post-migration HIV-acquisition. A transnational perspective on HIV prevention and sexual health promotion is needed for effectively reducing migrants’ HIV risk related to their mobility.

## Introduction

People immigrating from high endemic countries to Europe are highly affected by HIV: 18% of all newly diagnosed cases with known origin in the European Union/European Economic Area in 2017 came from high HIV prevalence areas [1]. Data from the European Surveillance System for 29 European countries estimated that Sub-Saharan African migrants (SAM) represented 53% of all HIV cases reported among people born outside the reporting countries, in 2007–2012 [2]. Belgium and Portugal, the two countries where this study was conducted serve as typical examples for European countries with colonial links now affecting migration patterns. Both countries have high proportions of SAM among their populations of people living with HIV. In Belgium, surveillance data from 2016 show that 30% of all new HIV infections with known origin were reported among SAM [3]. In Portugal, which has one of the highest HIV prevalence rates in western Europe, 18.4% of newly diagnosed HIV cases in 2017 were reported among people born in sub-Saharan Africa (accounting for 57.6% of foreign-born newly diagnosed HIV cases with known origin) [4].

Given the high HIV burden in SAM populations, understanding HIV transmission risks has been a focus of HIV research [5]. Against the background of increasing evidence on post-migration HIV acquisition among SAM in Europe, shown to be as high as 45% for SAM in a European multi-centre study [6], it is important to better understand the HIV transmission dynamics in this group [7,8].

Surprisingly, the role of post-migration mobility as a driver for HIV acquisition has received little attention [9] when compared to the available body of research on individual-level factors such as low risk perception [10], risky sexual behaviours [11], community-level factors such as sexual networks [12] or structural factors such as poverty [13]. Research has shown that experiences during the migration trajectories from resource-limited regions to European destination countries increase vulnerability to HIV acquisition [6]. In a society characterised by increasing mobility [14], migrants may also travel after having settled in host countries for several reasons, such as social connections or family ties, the search for work or documents. The available evidence shows that travelling is associated with having new sexual partners, engaging in risky sexual behaviours, and becoming part of local sexual networks [12,15,16]. For instance, a study among ethnic minorities living in Amsterdam revealed that participants were exposed to substantial HIV risk while visiting their homelands [17]. Similarly, a study among SAM living in London reported that about half of the sample had travelled back to their countries of origin in the past five years. Of these, 40% of men and 21% of women had sex with a new partner when travelling [18]. However, most studies to date have addressed migrants’ sexual behaviour only in the host country and have been confined to one city or country.

To inform evidence-based prevention planning for vulnerable populations such as SAM [19] obtaining reliable evidence on HIV acquisition risk requires a bio-behavioural data collection approach, including detailed information on mobility after settlement. Understanding the relationship between mobility and HIV risk among migrant populations in different European countries would allow for developing targeted national and transnational HIV policies and intervention programs to prevent new infections [20].

This study aimed to explore risky sexual behaviour and HIV-acquisition among SAM living in two European cities, and to assess their post-migration mobility as a determinant of sexual risk behaviour.

## Methods

This study draws on data from two cross-sectional bio-behavioural surveys conducted in Lisbon [21] and Antwerp [16] aiming to assess HIV prevalence, sexual risk behaviours and its determinants among SAM. Both bio-behavioural surveys adopted a similar community-based participatory research approach. Partner institutions and community-based organizations were deeply involved in every step of the study implementation. This assured the studies’ acceptability and facilitating access to migrant communities, as well as adapted data collection procedures and instruments. This approach was employed to allow for knowledge generation addressing communities’ health needs [22,23].

In both surveys, a venue-based sampling approach was used to recruit participants [24]. Based on formative research developed with community partners, a geographic and social mapping was conducted in both cities allowing for systematically identifying and listing venues usually frequented by SAM (e.g. bars, community associations, events and meetings of African organisations, shops, public places such as squares, parks and street corners). The Belgian survey used a two-stage time location sampling to recruit study participants. Sampling and recruitment procedures are described in detail elsewhere [16]. In brief, venues were selected with a probability proportion-to-size from the venues’ sampling frame. In case of unavailability, the next venue on the list was selected. At each venue, 14 participants present at the time of data collection were randomly selected. In the Portuguese survey, recruitment teams of outreach workers from partner associations attended the venues listed through the geographic and social mapping in the formative research and systematically approached eight to 12 participants inviting them to participate in the study. The methods and procedures used are described in detail elsewhere [25].

The inclusion criteria in the surveys were being ≥18 years old and reporting a sub-Saharan African country of origin. Data were collected through a questionnaire (on a tablet in Belgium and on paper in Portugal). The questionnaires comprised close-ended questions on sociodemographic and migration-related characteristics, mobility, sexual behaviour, self-reported HIV status, HIV testing and other sexually transmitted infections (STI). Sociodemographic characteristics included sex, age, country of birth, relationship status, educational level and employment status. Migration-related characteristics assessed were time living in the host country and immigration status. Participants were also asked about post-migration mobility, i.e., if they had travelled to an African country and/or to another European country after settling in their host country. Regarding sexual behaviour, data were collected on the number of sexual partners in the last year, country of last sexual intercourse, type and origin of last sexual partner, as well as condom use in the host country and abroad. After responding to the questionnaire, participants’ HIV status was assessed: in Belgium participants were asked to self-collect an oral fluid sample for HIV testing and in Portugal participants had a finger-stick whole-blood HIV-rapid test.

Ethical approval was obtained from the Institutional Review Board of the Institute of Tropical Medicine and the Ethical Committee of the University Hospital Antwerp for the Belgian survey, and from the Ethics Committee of Instituto de Higiene e Medicina Tropical for the Portuguese survey. All participants provided written informed consent before data collection. Confidentiality of data handling was guaranteed and emphasised when contacting the respondents to account for the sensitivity of the research topic. Data were pseudonymised to protect participants’ privacy and to ensure that participants could not be identified through the data collected.

### Data analysis

Datasets were merged and analysed using IBM SPSS Statistics 23.0. The current analysis only considered participants who reported ever having had sexual relations. Participants were first described in terms of their sociodemographic and migration-related characteristics, mobility, sexual behaviour, HIV infection, HIV testing and other STI. These characteristics were compared between participants who had and who had not travelled after settling in their host countries (“travellers” vs. “non-travellers”) using descriptive analysis and chi-square tests. In preliminary analyses, differences between participants traveling to African countries and those traveling to European countries were explored in terms of risky sexual behaviour, namely condomless sex and casual partner in the last sexual intercourse, as well as other main outcomes such as HIV testing and HIV status. However, no significant differences were found—participants who had travelled only to Africa (17.2%) and only to Europe (23.1%) did not significantly differ regarding condom use during the last intercourse (p = 0.488), type of partner in the last intercourse (p = 0.408), HIV testing ever (p = 0.090) and HIV status (p = 0.262). In addition, relatively small numbers of participants responded affirmatively to each variable (“travelled to an African country” and “travelled to a European country”), consequently limiting further statistical analyses. Therefore, the variables “travelled to an African country” and “travelled to a European country” were merged and a new variable was constructed: “having travelled abroad” defined “traveller” as people who, after settling in their host countries, had travelled outside that country for any reason. This allowed to explore differences between those travelling abroad (to both destinations) and those not travelling at all in terms of the outcome variables. Next, associations of condomless sex at last sexual intercourse with sociodemographic factors, mobility, sexual behaviour, HIV testing, self-reported HIV and STI were examined using multivariable logistic regression analysis. In order to analyse the use of condom accounting for known HIV status, a new variable was created combining the variables “ever had a HIV test” and “self-reported HIV status” into “HIV status based on the last HIV test”. The related response options were: “Positive status”, “Negative or unknown status” (unknown because results were not collected or received) and “Never tested for HIV”. In addition, the association between condomless sex in the host country and abroad was examined through multivariable logistic regression analysis. Country of survey was included as a variable in all models, regardless of significance. Post-migration sexual behaviour and sexual relationships abroad and in the host country among travellers were compared using Chi-square tests. Concurrency of sexual relationships was analysed based on reported engagement in overlapping sexual relationships with different partners during the previous year. This variable was descriptively analysed in the overall sample and in further exploratory analyses in participants who reported intercourse abroad while having a sexual partner in the country of residence during the last five years. Finally, HIV-positive participants’ mobility and sexual behaviour were explored using descriptive analysis. For all tests, statistical significance levels were set at <0.05. The magnitude of bivariate and multivariate associations was estimated by means of Odds Ratio and 95% confidence intervals calculated through logistic regression.

## Results

### Participants’ sociodemographic characteristics, mobility and sexual behaviour

Table 1 presents the characteristics of the total sample (N = 1508) which included 718 participants from Belgium (47.6%) and 790 participants from Portugal (52.4%). Male participants represented 58.0% of the total sample. Half of participants were between 30–49 years old and around a third were younger. Portugal had a larger proportion of participants older than 49 years than Belgium. Likewise, the majority had completed secondary education, but Belgium had a higher proportion of participants having completed higher education levels than Portugal. More Portuguese than Belgian study participants reported being non-employed. Half of the sample (50.5%) were living in the study country for more than 10 years, however, this proportion was larger among Portuguese compared to Belgian participants. Fourteen percent were of undocumented status, but this proportion was larger in Belgium than in Portugal.

**Table 1:** Characteristics of the total sample by study country and post-migration mobility.

	Total (N = 1508)		Belgian survey (47.6%, n = 718)		Portuguese survey (52.4%, n = 790)			Travelled abroad				
								Yes (68.4%, n = 1031)		No (31.6%, n = 477)		
	n	%	n	%	N	%	*p*-value	n	%	n	%	*p*-value
**Sociodemographics**												
Sex (n = 1508)												
Female	633	42.0	301	41.9	332	42.0	0.999	416	40.3	217	45.5	0.064
Male	875	58.0	417	58.1	458	58.0		615	59.7	260	54.5	
Age (n = 1508)												
18–29	517	34.3	251	35.0	266	33.7	<0.001	296	28.7	221	46.3	<0.001
30–49	749	49.7	411	57.2	338	42.8		545	52.9	204	42.8	
> 49	242	16.0	56	7.8	186	23.5		190	18.4	52	10.9	
Relationship status (n = 1479)												
In a relationship/married/civil partnership	730	49.4	417	60.5	313	39.6	<0.001	537	52.9	193	41.6	<0.001
Single/divorced/separated/widowed	749	50.6	272	39.5	477	60.5		478	47.1	271	58.4	
Educational level (n = 1490)												
Elementary education/no school education	333	22.3	123	17.6	210	26.6	<0.001	218	21.3	115	24.5	<0.001
Secondary education	864	58.0	329	47.0	535	67.7		574	56.2	290	62.0	
Higher education	293	19.7	248	35.4	45	5.7		230	22.5	63	13.5	
Employment status (n = 1411)												
Employed	603	42.7	321	51.7	282	35.7	<0.001	464	47.9	139	31.4	<0.001
Non employed	808	57.3	300	48.3	508	64.3		505	52.1	303	68.6	
Time living in the study country (n = 1448)												
Less than 5 years	405	28.0	282	42.9	123	15.6	<0.001	187	18.9	218	47.3	<0.001
Between 5 and 10 years	312	21.5	164	24.9	148	18.7		207	21.0	105	22.8	
More than 10 years	731	50.5	212	32.2	519	65.7		593	60.1	138	29.9	
Immigration status (n = 1507)												
Documented	1294	85.9	585	81.6	709	89.7	<0.001	930	90.2	364	76.5	<0.001
Undocumented	213	14.1	132	18.4	81	10.3		101	9.8	112	23.5	
**Sexual behaviours and relationships**												
Used condom with last partner (n = 1444)												
Yes	460	31.9	222	32.6	238	31.2	0.543	297	29.6	163	37.0	0.007
No	984	68.1	458	67.4	526	68.8		706	70.4	278	63.0	
Type of last partner (n = 1398)												
Regular	1035	74.0	504	78.3	531	70.4	0.001	727	74.8	308	72.3	0.354
Casual	363	26.0	140	21.7	223	29.6		245	25.2	118	27.7	
Origin of last partner (n = 1427)												
African	1038	72.4	504	73.7	534	71.9	0.442	720	73.1	318	71.9	0.653
Other	389	27.6	180	26.3	209	28.1		265	26.9	124	28.1	
Number of sexual partners during last year (n = 1508)												
<2	1214	80.5	578	80.5	636	80.5	0.999	830	80.5	384	80.5	0.999
≥3	294	19.5	140	19.5	154	19.5		201	19.5	93	19.5	
**HIV status, HIV testing and other STI**												
Self-reported HIV status (n = 1504)												
Positive	42	2.8	14	2.0	28	3.5	0.063	28	2.7	14	3.0	0.866
Negative/Don’t know	1462	97.2	700	98.0	762	96.5		1002	97.3	460	97.0	
Result of HIV test (n = 1491)												
Reactive	75	5.0	32	4.6	43	5.4	0.477	47	4.6	28	5.9	0.308
Negative	1416	95.0	669	95.4	747	94.6		972	95.4	444	94.1	
Ever had a HIV test (n = 1460)												
Yes	892	61.1	508	72.9	384	50.3	<0.001	627	62.4	265	58.2	0.147
No	568	38.9	189	27.1	379	49.7		378	37.6	190	41.8	
Self-reported STI (n = 1412)												
Yes	220	15.6	88	13.3	132	17.6	0.027	158	16.2	62	14.2	0.383
No	1192	84.4	574	86.7	618	82.4		817	83.8	375	85.8	

Overall, 68.4% of participants had travelled to other countries after having settled in the study country (Table 1). Of these, 41.0% had travelled to both Africa and within Europe, 25.2% had travelled back to Africa, and 33.8% had travelled to Europe (S1 Table). Compared to participants not travelling, those who did travel were significantly older, reported more frequently to be married or in a stable relationship, to have completed higher education, to be employed, to live in the host country for more than 10 years, and to have regular immigration status (Table 1).

In terms of sexual behaviour, participants who had travelled reported more frequently that their last sexual intercourse was condomless (Table 1). No significant differences were found between travellers and non-travellers regarding type of last partner, origin of last partner and number of sexual partners during last year.

### HIV infection, HIV testing and other STI

In the total sample, 2.8% (n = 42) reported to be HIV positive (Table 1): 3.8% of women and 2.1% of men (p = 0.045, S2A Table). Overall, 5.0% (n = 75) had a reactive HIV test result (6.7% of women and 3.8% of men, p = 0.012). The proportion of reactive results was similar among travellers and non-travellers (Table 1), and among those with documented compared to those of undocumented status (5.1% and 4.8%, respectively, p = 0.872) (S2B Table).

Of participants with a reactive result to the HIV test, 46.7% (n = 35) self-reported being HIV negative or not knowing their serostatus. Overall, 38.9% of participants had never been tested for HIV. A total of 15.6% reported ever having been diagnosed with a STI. No significant differences were found for HIV testing and reported STI between travellers and non-travellers, nor between participants with documented and undocumented status.

### Condomless last sexual intercourse

Of the participants for whom data on condom use was available (n = 1444), 68.1% (n = 984) had not used condoms with their last partner (Table 1). The multivariable logistic regression analysis (Table 2) showed that condomless sex was more often reported by women, participants aged 30 and above, travellers, those whose last sexual partner was regular, those who were never tested for HIV and those who reported a negative or unknown status based on their last HIV test (compared to those who reported having tested positive in their last HIV test).

**Table 2:** Factors associated with condomless last sexual intercourse.

	Crude OR (95% CI)	*p*-value	Adjusted OR (95% CI) *	*p*-value
Sex				
Male	1		**1**	
Female	**2.25 (1.78–2.86)**	**<0.001**	**1.89 (1.39–2.57)**	**<0.001**
Age				
18–29	1		**1**	
30–49	**1.68 (1.32–2.14)**	**<0.001**	**1.80 (1.33–2.43)**	**<0.001**
> 49	**2.20 (1.55–3.14)**	**<0.001**	**2.46 (1.53–3.98)**	**<0.001**
Educational level				
Primary education or less/no school	1		1	
Secondary or less	0.70 (0.52–0.93)	0.016	0.90 (0.61–1.33)	0.610
Higher education	0.74 (0.52–1.06)	0.100	0.68 (0.42–1.09)	0.112
Relationship status				
Single	1		**1**	
In a relationship	1.01 (0.81–1.27)	0.912	**1.33 (1.01–1.75)**	**0.045**
Country of survey				
Portugal	1		1	
Belgium	0.93 (0.75–1.16)	0.543	1.03 (0.76–1.39)	0.868
Travelled abroad (after settling in the host country)				
No	1		**1**	
Yes	**1.39 (1.10–1.76)**	**0.006**	**1.35 (1.01–1.81)**	**0.043**
Type of last partner				
Casual	1		**1**	
Regular	4.50 (3.48–5.81)	**<0.001**	**4.19 (3.09–5.69)**	**<0.001**
Origin of last partner				
Non-African	**1**		1	
African	**1.80 (1.41–2.30)**	**<0.001**	1.20 (0.89–1.62)	0.232
Three or more sexual partners over the last year				
No	**1**		1	
Yes	**0.45 (0.34–0.58)**	**<0.001**	0.84 (0.60–1.17)	0.307
HIV status based on the last HIV test				
Positive status	**1**		**1**	
Negative or unknown status	**2.91 (1.54–5.52)**	0.001	**5.11 (2.33–11.20)**	**<0.001**
Never tested for HIV	**3.42 (1.79–6.55)**	<0.001	**8.01 (3.58–17.95)**	**<0.001**
Self-reported STI				
Yes	1		1	
No	1.14 (0.83–1.55)	0.424	1.01 (0.71–1.45)	0.952

* Adjusted to all variables

Among travellers who reported sexual encounters abroad, condomless sex was high: 70.7% (n = 612) when the last intercourse was in the host country and 62.5% (n = 317) when it was abroad (Table 3). Almost half of travellers reported condomless sex in both settings (48.7%, n = 137), while 30.3% (n = 85) reported condomless sex in one of the settings, and 21.0% (n = 59) reported consistent protected sex (S3A Table).

**Table 3:** Sexual behaviour when travelling abroad and in the host country post-migration.

	Had intercourse abroad (49.2%, n = 507)		No condom use during the last intercourse abroad (62.5%, n = 317)			Had intercourse in the host country (84.0%, n = 866)		No condom use during the last intercourse in the host country (70.7%, n = 612)		
	n	%	n	%	*p*-value *	n	%	n	%	*p*-value *
Type of last partner										
Casual	191	39.1	77	41.4	**<0.001**	209	24.7	81	38.8	**<0.001**
Regular	297	60.9	235	79.9		637	75.3	518	81.3	
(missings)	(19)		(5)			(20)		(13)		
Origin of last partner										
African	406	83.4	270	67.5	**0.020**	617	72.2	460	74.6	**<0.001**
Other	81	16.6	42	53.2		237	27.8	147	62.0	
(missings)	(20)		(5)			(12)		(5)		

* Comparison between those who used condom and those who did not.

Condomless sex at last sexual encounter in the host country occurred more often among those who also reported no condom use abroad [OR: 5.32; 95% CI: 2.98–9.25] when controlled for gender, age, educational level, relationship status, country of the survey, type of partner, origin of last partner and number of sexual partners over the last year (S3B Table). Condomless sex was more frequent with a regular partner and with an African partner, both abroad and in the host country (Table 3). The proportion of condomless sex during the last intercourse in an African country was 65.6% (n = 231) and in an European country was 59.3% (n = 127). This was higher with regular partners (in Africa: 82.1% vs. 39.0% with casual partners; in Europe: 77.0% vs. 39.8% with casual partners), and with African partners (in Africa: 67.4% vs. 38.5% with non-African partners; in Europe: 64.1% vs. 53.9% with non-African partners) (S3C Table).

### Concurrent sexual relationships

Overall, engagement in concurrent sexual relationships in the previous year was more common among participants who had travelled abroad than those not travelling (28.9% vs. 23.8%, p = 0.048). The majority of participants who reported intercourse abroad during the last five years had a sexual partner in the host country and simultaneously, while being abroad (in Europe or in Africa), reported sexual encounters with another sexual partner. Also, around 39% of these participants reported concurrent sexual partnerships in the previous year. The last sexual partner abroad was either living abroad or was met there (i.e. 50.5% had a regular partner in the host country and a partner abroad; 27.7% had a casual partner in the host country and a partner met or living abroad) (Table 4). Most frequently, the last sexual partner in both host country and abroad was an African partner (73.3%), although a fourth reported that their last sexual partner was non-African.

**Table 4:** Partner types and their origins at last sexual intercourse (abroad and in the host country).

Last sexual partner in the host country	Last sexual partner abroad (in the last 5 years)*	N	%
Type of partner			
Regular (69.7%, n = 239)	Met abroad/living abroad	173	50.5
	Who travelled with	66	19.2
Casual (30.3%, n = 104)	Met abroad/living abroad	95	27.7
	Who travelled with	9	2.6
Engagement in concurrent sexual relationships	147	38.7
Origin of partner			
African (84.5%, n = 294)	African	255	73.3
	Non-African	18	5.2
Non-African (15.5%, n = 54)	African	39	11.2
	Non-African	36	10.3

* Sub-sample analysed: n = 380 (i.e. 75.0% of the 507 participants who had sex abroad). Missing data in type of partner = 37; missing data in origin of partner = 32.

### HIV-positive participants: sexual risk behaviour and mobility

Among the 75 participants with reactive HIV test-results, 39 reported condomless sex during their last sexual intercourse. More than half of them (n = 47) had travelled to other countries, and among them about half reported sexual intercourse abroad (n = 25) (Table 5). The majority of them did not report condom use with their last sexual partner abroad (n = 21). For most of them, this sexual partner was an African (n = 19) and a regular partner living abroad (n = 14) or who had travelled with them (n = 2). Over a half of those who had condomless sex abroad were unaware of their HIV-positive status (n = 10 reported not being HIV positive based on their last HIV test and n = 3 reported never having been tested). Among participants with reactive HIV test-results who did not report sexual intercourse abroad (n = 50), over a third (n = 19) did not use condom during their last intercourse in the host country (Table 5).

**Table 5:** Mobility and sexual behaviour of HIV-positive participants.

HIV-positive participants (n = 75)	Travelled to other countries (n = 47)	Had intercourse abroad (n = 25)	Did not use condom abroad (n = 21)	Type of partner when traveling (missing = 1) Casual partner, met abroad (n = 4) Regular partner, who lives abroad (n = 14) Regular partner, travelled with (n = 2)
				Origin of last partner when traveling (missing = 1) African (n = 19) Other (n = 1)
				Last intercourse was abroad (n = 7; 5 in Africa and 2 in Europe) Last intercourse was in host country (n = 14): - Used condom in last intercourse in host country (n = 4) - Did not use condom in last intercourse in host country (n = 10)
				Never tested for HIV (n = 3) Negative or unknown status, based on the last HIV test (n = 10) Positive status, based on the last HIV test (n = 8)
			Used condom abroad (n = 4)	Type of partner when traveling Casual partner, met abroad (n = 1) Regular partner, who lives abroad (n = 1) Regular partner, travelled with (n = 2)
				Origin of last partner when traveling African (n = 4)
				Last intercourse was abroad (n = 2; 1 in Africa and 1 in Europe) Last intercourse was in host country (n = 2): - Used condom in last intercourse in host country (n = 2)
				Positive status, based on the last HIV test (n = 4)
		Did not have intercourse abroad (n = 22)	Last intercourse was abroad (n = 3) Last intercourse was in host country (n = 19): - Used condom in last intercourse in host country (n = 11) - Did not use condom in last intercourse in host country (n = 8)	
	Did not travel to other countries (n = 28)	Last intercourse was abroad (n = 5) Last intercourse was in host country (n = 22): - Used condom in last intercourse in host country (n = 11) - Did not use condom in last intercourse in host country (n = 11)		

## Discussion

This bio-behavioural study explored the role of mobility in sexual risk taking and HIV acquisition among SAM residing in two European cities. Two thirds of the participants travelled to other countries after having settled, and for two thirds among them their destinations included African countries. The main findings show that SAM who travelled post-migration are at increased risk for HIV, reporting more condomless sex and concurrency than non-travellers, although engagement in sexual risk behaviours was found in the overall sample. HIV prevalence among the studied sample was 5% and a high proportion of 39% had never tested for HIV.

These findings on SAMs’ mobility are in line with available research showing that migrants who become settled in host countries, visit their origin home countries and other destinations, whether for domestic, conjugal, social or economic purposes [17, 18]. The high HIV prevalence in this study points to a substantial HIV risk exposure in the studied samples, corroborating recent research on post-migration HIV acquisition in Europe [6, 13]. Research conducted in Africa has also shown that migrants adopt risky sexual behaviour while away from their home country compared to non-migrants [26–28]. In Europe, such specific studies are largely lacking, however, the few available studies showed that migrants’ mobility was associated with increased risk for HIV [17, 18, 29].

HIV risk while travelling is associated with individual risk behaviour: about two thirds of the travellers (63%) in our sample did not use a condom when having sex abroad. The use of condomless sex as an indicator for sexual risk behaviour could be discussed. It is important to understand factors such as relationship quality and context, sexual partner’s serostatus or uptake of other prevention means such as PrEP. However, while theoretically condomless sex may be seen not as a risk factor in long-term monogamous relationships, most HIV transmissions occur in the context of long-term relationships [30, 31], with a great body of evidence showing that many individuals in such relationships report sexual non-exclusivity and multiple sexual partners [32, 33]. PrEP uptake among heterosexual migrants in Europe so far has been extremely low [34,35]. Against this background, condomless sex in small sexual networks with high proportions of late diagnoses [36] may indeed serve as a relevant outcome. The odds of not using a condom in the host country was five times higher when the last sexual intercourse abroad was also condomless. This may be associated with patterns of condomless sex, as 48.7% of travellers reported condomless sex both in host countries and abroad. It may indicate habit-forming of not using condoms [37], thus explaining risk behaviour when travelling. It shows the need for tailored prevention messages highlighting individual risk related to prevention behaviours, both in home and destination countries, as well as HIV prevention strategies on transnational levels.

In this study, three in four travellers reported that their last sexual partners in the host country and abroad were Africans, which is in line with previous research on assortative sexual mixing [38, 39]. Studies from the Netherlands and France showed that up to 80% of participants reported assortative sexual mixing in intra-African sexual networks [12, 40]. Other research on sexual mixing between high- and low-risk populations reported assortative mixing based on demographic rather than sexual characteristics [41]. Nevertheless, our study shows that dissortative sexual mixing also occurs, since 26.7% of the participants reported both African and non-African sexual partners.

About half of the travellers reported concurrency, mostly having a regular partner in the host country and other sexual partners abroad. In Belgium, this has been referred to as the culturally well-accepted “deuxieme bureau”, for instance among the Congolese community [42]. Condomless sex happened more often with a regular and an African partner indicating trust as a barrier to condom use within sexual relationships [43, 44]. Concurrent partnerships potentiate the spread of HIV by expanding sexual network size, enhancing network connectivity, and increasing rate and efficiency of disease transmission before individuals are aware of their infection [45, 46]. Our findings on concurrency show the fluid meaning of “regular partners”, which may not always be aligned with the definition usually adopted in epidemiological research. HIV prevention messages thus should consider culturally grounded sexual norms and their influence on individual risk perception. The latter has been described as low among African migrants [10] who therefore may have little demand for HIV prevention [47].

Overall, the proportion of condomless sex among the study sample was high (68.1%), corroborating high levels of risky sexual behaviours among migrant populations found in other research [48–50]. In our study, specific risk factors associated with condomless sex included being female, being ≥30 years old, having travelled, having had the last sexual encounter with a regular partner, having never been tested for HIV, and having a non-reactive test-result in the study. Epidemiological evidence shows that HIV risk for women is often associated with regular relationships, since interpersonal dynamics in long term relationships may act as a barrier to adopting protective behaviours [51]. Alongside, migration-related stressors including adaptation processes in host countries have been viewed as increasing migrants’ vulnerability for sexual health-related problems, such as HIV and other STI [52]. By having condomless sex in both their host country and their homeland, migrants may be at risk of cross-border transmission of HIV and other STI as documented elsewhere [53]. A study with travellers visiting clinics of a worldwide sentinel surveillance network found that travelling to visit friends and relatives was associated with diagnosed STI [54].

Belgium and Portugal are both countries with concentrated HIV epidemics with large proportions of migrant populations affected. The overall HIV prevalence found among the studied sample was five percent based on reactive HIV test result. This was significantly higher among women (6.7% compared to 3.8% among men). This reflects patterns of generalised HIV epidemics in SSA regions, as found in other European countries [55].

Currently, the identification of undiagnosed HIV is both a priority and a challenge to ensure the HIV continuum of care [56]. Our study confirms that this may indeed be the weakest stage of the HIV cascade as our analysis revealed a high proportion of participants not knowing or not reporting their HIV status. The overall rate of 46.7% indicates that potentially one in two individuals could be undiagnosed. In addition, we found a high degree of participants who never tested for HIV: 27.1% in Antwerp and 49.7% in Lisbon. While similar results were described in other studies [47, 57, 58] potential under-reporting of known HIV status should also be considered. Our analysis showed particular high rates of unreported/undiagnosed HIV among women (i.e. 54.3%), especially among those of undocumented status. This may be surprising given that prenatal HIV screening offers constitute HIV testing opportunities for women in both countries. However, a recent study using mathematical modelling in Belgium showed that indeed women of sub-Saharan African origin have a high risk of being undiagnosed [59]. Migration status may be one of the structural factors impeding access [60, 61], but future research should explore this finding in more detail. HIV screening programs should consider this policy-practice gap, and invest more in further reducing thresholds to health care and HIV testing through tailored community-based initiatives [62] or in primary-care settings [63], in particular for women and those being undocumented.

Finally, our study indicates a considerable risk of onwards HIV transmission as highlighted by the high rate of unknown or unreported HIV status along with the occurrence of condomless sex associated to mobility among HIV-positive participants (assuming that those who knew their status were on treatment and thus could not transmit HIV). Secondary prevention to reduce sexual risk behaviours among HIV-positive migrants should be improved within overall efforts to promote knowledge of HIV-status and minimise transmission risk through timely treatment.

This study’s strength lies in using data from two carefully designed, bio-behavioural surveys conducted in SSA community settings with a strong participatory research component. It resulted in comparable behavioural data for more than 1500 SAM living in two European cities. So far, this constitutes a unique approach in Europe. However, limitations must be acknowledged. The bio-behavioural surveys used were originally designed to assess HIV prevalence. The samples differ in terms of participants’ ethnic origins. The observed heterogeneity reflects the historic links of Belgium and Portugal with sub-Saharan Africa due to their colonial past. We could not capture any cultural specificities and associated sexual norms and practices, which should be further investigated in future research. Using a set of common comparable core-variables further limited the potential covariates. Therefore, mobility could only be explored but not investigated in-depth. This paper focused only on international travel (including travels within and outside of Europe), not collecting information on smaller-scale mobility. Future research should also investigate the link between HIV risks and such mobility patterns, such as rural-urban movements on the search for work and better living conditions. Post-migration travel can be correlated with age and time in the country, two acknowledged factors for increased HIV risk [64–66], which may partially explain the association found between post-migration travel and HIV risk. Nevertheless, these findings highlight the need for prevention by supporting migrants in forming the habit of taking preventive measures when having sex abroad, especially among older and long-time resident migrants. Further behavioural information such as gender of sexual partners or type of sex (vaginal/anal) would be interesting to investigate in the context of risk and mobility. However, in both studies a community-based participatory research approach was applied throughout all the steps of the study including its conceptualization, in which the perspectives and concerns of the communities regarding conducting a survey on sensitive topics were taken into account. Besides community partners as members of community advisory boards, also community researchers who had insider knowledge, recommended not to ask sensitive questions, as many participants would not be willing to report socially undesirable behaviours. Therefore, it was agreed by the project partners that no information such as sexual relations with same-sex partners and type of sex (vaginal/anal), that could be perceived as intrusive, would be collected. Self-reported data, in particular on sexual behaviour and HIV status, may have resulted in bias.

Despite the limitations, some meaningful conclusions with relevance for prevention can be drawn: many SAM are mobile after settling in their host countries, and obviously acquired patterns of sexual risk behaviour may not change when people travel. Sexual health promotion and HIV prevention strategies should thus incorporate specific travel advice, such as reinforcing condom promotion (including female condoms) and sexual risk reduction. Additional new prevention methods, i.e. Pre-Exposure Prophylaxis (PrEP), which so far has not been taken up by this group [67, 68] should also be promoted within combination prevention [34]. Mobile populations could benefit in particular from innovative biomedical prevention technologies in the pipeline such as long acting PrEP and HIV treatment, and delivery of these technologies to be easily accessible to community settings [69].

Our study shows the high need for promotion of HIV testing among mobile populations, which can be achieved through a mix of strategies, such as promotion of community-based HIV testing, self-testing and sampling [70] and provider-initiated HIV testing with consideration of human rights and gender aspects. The available European evidence should guide intensified efforts to reduce the proportion of undiagnosed HIV among mobile and migrant populations and ensure linkage to care [71].

This study has provided a first exploration of mobility as a driver of the HIV epidemic within mobile SSA migrant populations residing in Europe. Future studies employing mixed methods research should be conducted to provide a better understanding of the role of post-migration mobility in HIV risk. Such knowledge can inform HIV prevention and sexual health promotion in a transnational perspective with impact in both host countries and abroad.

## Supporting information

S1 File Data base. (SAV)

S1 Table Destination of travel of participants who travelled to other countries after having settled in the study country. (DOCX)

S2 Table Additional information on self-reported HIV status and HIV rapid test results. (DOCX)

S3 Table Additional information on condomless sex. (DOCX)
