# Supplementary material for: The role of mobility in sexual risk behaviour and HIV acquisition among sub-Saharan African migrants residing in two European cities
Source: PLoS One. 2020 Feb 5;15(2):e0228584. doi: 10.1371/journal.pone.0228584 (PMC7001961; doi:10.1371/journal.pone.0228584)
Supplement: S1 Table — (DOCX) [file pone.0228584.s002.docx]

S1 Table – Destination of travel of participants who travelled to other countries after having settled in the study country (n=1031).

|  | **n** | **%** |
| --- | --- | --- |
| **Destination of travel after having settled in the study country** |  |  |
| Africa | 260 | 25.2 |
| Europe | 348 | 33.8 |
| Both Africa and Europe | 423 | 41.0 |
