# Supplementary material for: The role of mobility in sexual risk behaviour and HIV acquisition among sub-Saharan African migrants residing in two European cities
Source: PLoS One. 2020 Feb 5;15(2):e0228584. doi: 10.1371/journal.pone.0228584 (PMC7001961; doi:10.1371/journal.pone.0228584)
Supplement: S2 Table — (DOCX) [file pone.0228584.s003.docx]

S2A Table - Self-reported HIV status across sex.

|  | **HIV positive**  **(self-reported) (n=42; 2.8%)** | | **HIV negative/don’t know**  **(self-reported)**  **(n=1462; 97.2%)** | |  |
| --- | --- | --- | --- | --- | --- |
|  | **n** | **%** | **n** | **%** | **p-value** |
| **Sex** |  |  |  |  |  |
| Female | 24 | 3.8 | 609 | 96.2 | 0.045 |
| Male | 18 | 2.1 | 853 | 97.9 |  |

S2B Table - HIV rapid test result across sex and immigration status.

|  | **Reactive result to HIV test (n=75; 5.0%)** | | **Negative result to HIV test**  **(n=1416; 95.0%)** | |  |
| --- | --- | --- | --- | --- | --- |
|  | **n** | **%** | **n** | **%** | **p-value** |
| **Sex** |  |  |  |  |  |
| Female | 42 | 6.7 | 586 | 93.3 | 0.012 |
| Male | 33 | 3.8 | 830 | 96.2 |  |
| **Immigration status** |  |  |  |  |  |
| Regular | 65 | 5.1 | 1217 | 94.9 | 0.872 |
| Undocumented | 10 | 4.8 | 198 | 95.2 |  |
