# Supplementary material for: The role of mobility in sexual risk behaviour and HIV acquisition among sub-Saharan African migrants residing in two European cities
Source: PLoS One. 2020 Feb 5;15(2):e0228584. doi: 10.1371/journal.pone.0228584 (PMC7001961; doi:10.1371/journal.pone.0228584)
Supplement: S3 Table — (DOCX) [file pone.0228584.s004.docx]

S3A Table – Condomless sex during last intercourse among participants who have travelled abroad and reported sexual encounters.

|  | **n** | **%** |
| --- | --- | --- |
| **Condomless sex in the last intercourse** |  |  |
| Yes, in both host country and abroad | 137 | 48.7 |
| Yes, only in the host country or only abroad | 85 | 30.3 |
| No | 59 | 21.0 |

S3B Table – Association between condomless sex at last sexual encounter in the host country and condomless sex at last sexual encounter abroad.

|  | **Condomless sex at last intercourse in the host country** | | | |
| --- | --- | --- | --- | --- |
|  | **Crude OR**  **(95% CI)** | **p-value** | **Adjusted OR ***  **(95% CI)** | **p-value** |
| **Condom use at last intercourse abroad**  Yes  No | 1  3.52 (2.26-5.49) | <0.001 | 1  5.32 (2.98-9.25) | <0.001 |
| * Adjusted for gender, age, educational level, relationship status, country of the survey, type of partner, origin of last partner and number of sexual partners over the last year. | | | | |

S3C Table – Condom use in the last sexual intercourse in Africa and in the last sexual intercourse in Europe, including by type and origin of partner.

|  | **Last sexual intercourse in Africa (n=361)** | | | |  | **Last sexual intercourse in Europe (n=223)** | | | |  |
| --- | --- | --- | --- | --- | --- | --- | --- | --- | --- | --- |
|  | **Condom use** | | **No condom use** | | **p-value** | **Condom use** | | **No condom use** | | **p-value** |
|  | **n** | **%** | **n** | **%** |  | **n** | **%** | **n** | **%** |  |
| **Total** | 121 | 34.4 | 231 | 65.6 |  | 87 | 40.7 | 127 | 59.3 |  |
| (missings) | (9) | | | |  | (9) | | | |  |
| **Type of partner** |  |  |  |  |  |  |  |  |  |  |
| Casual | 75 | 61.0 | 48 | 39.0 | <0.001 | 59 | 60.2 | 39 | 39.8 | <0.001 |
| Regular | 39 | 17.9 | 179 | 82.1 |  | 26 | 23.0 | 87 | 77.0 |  |
| (missings) | (20) | | | |  | (12) | | | |  |
| **Origin of partner** |  |  |  |  |  |  |  |  |  |  |
| African | 109 | 32.6 | 225 | 67.4 | 0.031 | 46 | 35.9 | 82 | 64.1 | 0.153 |
| Other | 8 | 61.5 | 5 | 38.5 |  | 35 | 46.1 | 41 | 53.9 |  |
| (missings) | (14) | | | |  | (19) | | | |  |
